# A genome‐wide analysis suggests pleiotropic effects of Green Revolution genes on shade avoidance in wheat

**DOI:** 10.1111/eva.13349

**Published:** 2022-02-19

**Authors:** Michel Colombo, Germain Montazeaud, Véronique Viader, Martin Ecarnot, Jean‐Marie Prosperi, Jacques David, Florian Fort, Cyrille Violle, Hélène Fréville

**Affiliations:** ^1^ AGAP, Univ Montpellier, CIRAD, INRAE, Institut Agro Montpellier France; ^2^ CEFE, Univ. Montpellier, CNRS, EPHE, IRD Montpellier France; ^3^ 27213 Department of Ecology and Evolution University of Lausanne Lausanne Switzerland

**Keywords:** durum wheat, evolutionary prebreeding population, light quality, phenotypic plasticity, plant height, QTL

## Abstract

A classic example of phenotypic plasticity in plants is the suit of phenotypic responses induced by a change in the ratio of red to far‐red light (R∶FR) as a result of shading, also known as the shade avoidance syndrome (SAS). While the adaptive consequences of this syndrome have been extensively discussed in natural ecosystems, how SAS varies within crop populations and how SAS evolved during crop domestication and breeding remain poorly known. In this study, we grew a panel of 180 durum wheat (*Triticum turgidum* ssp. *durum*) genotypes spanning diversity from wild, early domesticated, and elite genetic compartments under two light treatments: low R:FR light (shaded treatment) and high R:FR light (unshaded treatment). We first quantified the genetic variability of SAS, here measured as a change in plant height at the seedling stage. We then dissected the genetic basis of this variation through genome‐wide association mapping. Genotypes grown in shaded conditions were taller than those grown under unshaded conditions. Interaction between light quality and genotype did not affect plant height. We found six QTLs affecting plant height. Three significantly interacted with light quality among which the well‐known Rht1 gene introgressed in elite germplasm during the Green Revolution. Interestingly at three loci, short genotypes systematically expressed reduced SAS, suggesting a positive genetic correlation between plant height and plant height plasticity. Overall, our study sheds light on the evolutionary history of crops and illustrates the relevance of genetic approaches to tackle agricultural challenges.

## 1 INTRODUCTION

Competition for light is one of the main factors shaping plant–plant interactions and plant community structure. In natural ecosystems, such competition has led to the evolution of the shade avoidance syndrome (SAS; Schmitt & Wulff, 1993), whereby plants display morphological and physiological plasticity in response to a decrease in light quantity and changes in light quality induced by neighboring plants (Holmes & Smith, 1975; Smith, 1982). Indeed, as leaf chlorophyll absorbs more red‐light (R, 635–700 nm) than far‐red light (FR, 700–800 nm), and green tissues reflect FR light, the R:FR ratio decreases in the presence of other plants, which triggers the SAS. Plant perception of R:FR is mediated by phytochromes (Ballaré et al., 1990), which triggers a suit of phenotypic changes in response to lower R:FR, including stem elongation, early flowering, reduced branching, reduced biomass, increased height, decreased leaf number, higher specific leaf area, lower chlorophyll a/b ratio, and decreased photoassimilation rates (Carriedo et al., 2016; Franklin, 2008; Holt, 1995; Sawers et al., 2005; Smith et al., 1992; Smith & Whitelam, 1997). Shade avoidance occurs mainly in the canopy of ecosystems where competition for light is the strongest. For instance, SAS has been well documented in many herbaceous species adapted to sunny conditions (e.g., *Arabidopsis thaliana*: Crepy & Casal, 2015; Bongers et al., 2018; *Impatiens capensis*: McGoey & Stinchcombe, 2009; *Datura ferox* and *Sinapis alba*: Ballaré et al., 1990). In forest habitats, SAS has been described among trees (Henry & Aarssen, 2001a; Peer et al., 1999), while attenuated in the undergrowth layer (Dudley & Schmitt, 1995). SAS has been shown to be an adaptive plastic response of sun‐exposed plants in natural ecosystems in many species (Dudley & Schmitt, 1995).

In agriculture, where plants are grown at high density, competition for light can strongly reduce crop productivity (Donald, 1968). Competitive phenotypes are indeed those that are able to outcompete their neighbors by investing more in resource‐harvesting organs at the expense of offspring production, leading to a negative correlation between individual competitiveness and seed production of the group, a so‐called tragedy of the commons (Anten & Vermeulen, 2016; Hardin, 1968). Empirical work of agronomists (Jennings & de Jesus, 1968), theoretical work using evolutionary game theory (Anten & Vermeulen, 2016 for a review), and kin selection theory (Montazeaud et al., 2020) have well documented such a negative correlation for plant height when plants compete for light. Indeed, groups of tall plants have a high resource investment in resource harvesting organs at the expense of seed production, resulting in strong competitive interactions among plants and low seed production. Reducing the negative effects of competition for light on yield at high planting densities has thus become a key challenge for breeders since the Green Revolution (Donald, 1968). In this line, the crop ideotype has been defined as a weak competitor with phenotypic characteristics such a short stems and few erect leaves, limiting community‐wide resource depletion (Donald, 1968). In agreement with this idea, the introduction of reduced height alleles (Rht) to limit lodging at high nitrogen supply led to a drastic decrease of plant size and an increase in yield in wheat and rice (Hedden, 2003). Increase in height is one of the most well‐known responses of plants to shading conditions as it leads to enhanced access to light (Holmes & Smith, 1975). Such plastic response to shading conditions results in taller plants and thus strongest competitive interactions among plants, lowering seed productivity (Fréville et al., 2019). Therefore, it has been suggested that SAS‐mediated height plasticity might lead to a tragedy of the commons (Kebrom & Brutnell, 2007a).

In natural communities, it has been shown that plants expressing SAS can suffer from strong fitness costs, especially in low resource ecosystems where there is a high risk of carbon balance deficit, and water or nutrients exhaustion (Ganade & Westoby, 1999; Grime & Mackey, 2002). Therefore, SAS‐mediated tragedy of the common might only occur in high‐input agrosystems, where the cost of plasticity is outweighed by the fitness benefit of escaping light competition. In this context, one could argue that SAS has been selected against to improve crop yield (Carriedo et al., 2016; Kebrom & Brutnell, 2007a; Smith et al., 1992; Wille et al., 2017). However, years after the beginning of the Green Revolution in the 1960s and the spread of high‐input agrosystems, many cereal crops still display shade avoidance responses (Casal et al., 1996; Kebrom & Brutnell, 2007a; Maddonni et al., 2002; Morgan et al., 2002). Thus, a more parsimonious hypothesis could be that some phenotypic responses of the syndrome that trigger yield loss might have been attenuated during the evolutionary history of crops, while others that increase yield might have been selected (Kebrom & Brutnell, 2007a). For instance, the SAS mediated by branch elongation has been attenuated in maize, where the teosinte allele of the teosinte branched1 (*TB1*) gene introgressed in a modern maize background, induces greater phenotypic plasticity in response to light than the maize allele (Lukens & Doebley, 1999). In contrast, sunflower plants are able to modify stem angles to incline away from neighbors in high‐density stands in response to changes in light quality, and this spatial re‐organization increases yield (Pereira et al., 2017). Overall, it remains challenging to assess the agronomic consequences of shade avoidance in crops and therefore to evaluate the potential interest of breeding on this trait.

Exploring SAS variations and their underlying genetic determinism in cultivated species could be promising to shed light on ecological processes at play during plant domestication and breeding and to target relevant genotypes for agriculture. This is all the more important today as introducing diversity in plant breeding programs is becoming a key challenge for promoting sustainable agriculture (Barot et al., 2017; Litrico & Violle, 2015), and selecting for phenotypes adapted to heterogenous competitive habitats is critical in that context. Genetic variability is a prerequisite for selection to operate on SAS‐mediated traits. The molecular bases of SAS have been well characterized in model species (Courbier & Pierik, 2019; Fernández‐Milmanda & Ballaré, 2021; Kebrom & Brutnell, 2007b; Sessa et al., 2018), but its intraspecific genetic variability has not been well documented. In wheat, one study found contrasted tillering responses to low R:FR between genotypes (Casal, 1988). However, the experimental design was not adapted to discuss the results from an evolutionary perspective.

Using 180 inbred lines sampled from a highly diversified evolutionary prebreeding population founded with wild, primitive, and cultivated wheat (David et al., 2014), we first assess genetic variability of plasticity of plant height and test for association between SAS and allelic variation at a set of 46,439 markers. We then discuss our results in light of the evolutionary of crops during domestication and breeding.

## 2 MATERIAL AND METHODS

### 2.1 Genetic material and genotyping

We used 180 inbred lines from the highly diversified Evolutionary Prebreeding durum wheat pOpulation (EPO) developed since 1997 at INRAE Montpellier, France (David et al., 2014). This genetic material was generated from twelve generations of open pollination and intercrossing between a large number of accessions from wild, primitive, and elite subspecies of *Triticum turgidum*. Allogamy was promoted by maintaining a male sterility recessive nuclear gene to obtain 10% of outcrossing at each generation (David et al., 2014). In 2009, lines were extracted from the 12th EPO generation and underwent two successive generations of selfing by single seed descent. This design led to a weak linkage disequilibrium (LD) structure, making this panel ideal for Genome‐wide Association Studies (GWAS). The EPO panel was genotyped with the TaBW410K marker high‐throughput genotyping array (Rimbert et al., 2018) from which marker and presence/absence variants (Off Target Variants) were extracted. Durum wheat is an allotetraploid species, which combines two independent diploid genomes A and B, 7 chromosome pairs each (2*n* = 4X = 28). Since polymorphic markers were biallelic, each genotype could carry 0, 1, or 2 copies of the most frequent allele, hereafter referred to as the reference allele. Nonpolymorphic markers and markers with a minor allele count under 10 were discarded. Missing values were imputed as the observed allele frequencies at each locus. After these steps, 46,439 markers were available for further analysis.

### 2.2 Growth conditions and phenotyping

Seeds were sown in 4 cm wide and 6 cm deep micropots, with two seeds per micropot and 60 micropots per tray. Micropots were kept in the dark at 8°C for 11 days to trigger germination. Out of the two plants per micropot, we only kept the one showing a coleoptile around 1.5 cm tall to start the experiment with homogeneous plant height. Plates were then transferred in phytotrons during 13 days at 20°C with 12:12 day:night cycles and sufficient watering. Phytotrons were kept closed during the course of the experiment to avoid any change in light quantity and quality. We used two phytotrons, one for each light quality. Light was produced with three kinds of colored LEDs (blue, far‐red, and red). In the unshaded treatment, only red LEDs were lighted at 100% of their intensity, leading to a high R‐FR ratio of 7. In the shaded treatment, only far‐red and white LEDS were lighted at 100% of their intensity, leading to a low R‐FR ratio of 0.3. R‐FR ratios were measured in each treatment with a spectrometer (HR4 from OceanOpics in the 350–1050 nm range). Red corresponds to the photon irradiance between 660 and 670 nm, and far red corresponds to photon irradiance between 725 and 735 nm. R:FR ratio was computed by the ratio of the sum of irradiances between 660 and 670 nm to the sum of irradiances between 725 and 735 nm. PAR was computed as the sum of photosynthetically photon flux density (PPFD) between 400 and 700 nm. PPFD at each wavelength was computed following Wang et al. (2021):

$$\text{PPFD}=\frac{\text{Irr}\times \text{lambda}}{\text{Na}\times h\times c}$$

where Irr is the irradiance, lambda is the wavelength, Na is the Avogadro constant, h is the Planck constant, and c is the speed of light.

In both treatments, light intensity in the photosynthetically active radiation (PAR) range was kept constant at approx. 250 µmol m^−2^ s^−1^. This allowed us to test only the effect of light quality independently from the effect of light intensity on plant growth. Controlling for light intensity is of high importance for two reasons. First, changes in the R:FR ratio affect plant growth well before neighboring plants start to shade each other (Ballaré et al., 1990). Second, light intensity‐related processes involve other molecular pathways than R:FR‐related processes (Hersch et al., 2014). Light quality and intensity were controlled by adjusting the intensity of the LEDs. Based on preliminary tests, we put the plants 60 cm away from the LEDs. This distance was indeed found to be optimal to ensure homogeneous light quantity and quality over the whole array of micropots.

Each genotype was replicated 4 times in each treatment. Because each phytotron could hold 2 micropot trays (120 plants), we replicated the experiment 6 times with a subset of the 180 genotypes in each replicate. Replicates were split randomly over the 6 batches to avoid confounding effects between batch and genotype. To avoid phytotron light quality confounding effects, phytotrons used for each light quality were reversed between batches.

We measured the initial height of each plant (H_i_) as the length of the coleoptile right before transferring trays into phytotrons. At the end of the experiment, plants had 2–3 leaves with no stem. We measured final plant height (H_f_) by measuring the longest leaf when plants were 24 days old.

### 2.3 Statistical analysis

All statistical analyses were performed with R v. 3.5.3 (R Core Team, 2019).

#### 2.3.1 Phenotypic analyses

We used a general linear model with final plant height (H_f_) as a response variable, initial height (H_i_) as a covariate, and batch, genotype, light quality, and interactions between light quality and genotypes as fixed effects. As we had many repetitions (4 per genotype per light quality) and only few batches (6), all effects were considered as fixed. This enabled us to compare the size of all effects. This would not have been possible using models with both random and fixed effects. We used the *lm*() and the *anova*() functions to perform these analyses. Fixed effects were tested using incremental F‐tests with a 5% significance level. To characterize the proportion of the variability explained by each response variable, we computed η² as the ratio of the sum of squares of the tested effect to the total sum of squares. We estimated broad‐sense heritability in each light condition (H²), by computing the proportion of phenotypic variation explained by genetic variation as follows:

$$H^{2}=\frac{\sigma_{g}^{2}}{\sigma_{g}^{2}+\sigma_{e}^{2}}$$

with $\sigma_{g}^{2}$ the genetic variance due to the genotype effect and $\sigma_{e}^{2}$ the residual variance due to all other effects. BLUE (best linear unbiased estimators) of genetic values, that is, accounting for both the effects of genotype and genotype by light quality interaction, were extracted for each genotype to perform GWAS analysis. Since the genetic variance depends upon the magnitude of the measuring units of the traits, the coefficient of variation (CV) of BLUE was used as a measure of the genetic variability independent of trait units. CV was computed for each light condition as σ_g_ / *μ*, with *μ* the mean of observations.

#### 2.3.2 Genomic analyses

We used a Genome‐Wide Association Study (GWAS) to identify the genomic regions associated with plant height plasticity in response to light quality. We could not directly test the marker × light quality interaction effect with a classical genome‐wide sequential type I test because of huge inflation in the QQ‐plot, a well‐known issue in GxE studies (Ueki et al., 2019; Voorman et al., 2011). Thus, to perform the GWAS, we used a 3‐step approach.

First, we fitted a linear mixed model for each marker where we jointly tested the marker and marker × light quality effects on final plant height. This model included a genomic relationship matrix K to control for genetic structure as described below. The joint effect of marker and marker × light quality was termed the “genetic effect.” Second, we conducted a clustering analysis to group all significant markers into quantitative traits loci (QTLs). Third, to disentangle the marker main effects from the marker x light quality effects for each detected QTL, we performed a sequential Wald F‐test and computed the size effect of the marker (β_marker_) and the size effect of the interaction between the marker and light quality (β_inter_).

In a first step, we fitted the following mixed model for each marker (Yu et al., 2006):

$$y=X\beta +Z_{1}u+Z_{2}u_{e}+\varepsilon$$

where **y** is the vector of 360 BLUE genetic values of final plant height (180 genotypes x 2 light conditions), **X** represents the incidence matrix for the vector of fixed effects **β** including intercept, light quality, marker, and marker x light quality, **Z_1_** and **Z_2_** are the incidence matrices for random effects associated with **u** (the vector of random polygenic effect) and **u_e_** (the vector of random polygenic genotype by light quality interaction effect), respectively, and ε is the vector of residual errors. Variances of random effects can be estimated as follows:

$$\text{Var}\left(u\right)=K\sigma_{u}^{2},\;\text{Var}\left(\varepsilon \right)=I\sigma_{e}^{2},\;\text{Var}\left(u_{e}\right)=Z_{1}{\mathrm{KZ}}_{1}^{t}{\mathrm{xZ}}_{E}Z_{E}^{t}\sigma_{\text{ue}}^{2}$$

where **K** represents a matrix of genetic relatedness between individuals (see below), I is the identity matrix, and **Z_1_** and **Z_2_** are described above. **Z_E_** is an incidence matrix for environmental effects used only to compute **Z_2_**. σ²_u_ and σ²_e_ are the polygenic and error variances, respectively. **^t^Z** denotes the **Z** transposed matrix. **K** was computed following (VanRaden, 2008) equation:

$$K=\frac{{\mathrm{VV}}^{\prime }}{2\sum \text{pi}(1‐\text{pi})}$$

with **V**, the centered marker matrix and pi the allele frequency at marker i.

Each marker was tested successively with the function *MMEst()* from the package *MM4LMM* (Hunter & Lange, 2004). The *AnovaTest()* function of this same package was used to evaluate the significance of markers for the test of the genetic effect and the sequential type 1 test. To test for the association between variation in plant height and allelic variation at each marker, we used a false discovery rate (FDR) approach to account for multiple testing (one test per marker) of 5% (Benjamini, 2010; Benjamini et al., 2001). This approach has been widely used in GWAS studies in place of the Bonferroni correction known to be over‐conservative (Brzyski et al., 2017).

In a second step, to group all significant markers into QTLs, we followed the method inspired from Cormier et al. (2014). We used the *R* ^2^ estimator (Hill & Robertson, 1968) to assess linkage disequilibrium (LD) between significant markers belonging to the same chromosome. LDs were then square‐root‐transformed to approximate a normally distributed random variable (Breseghello & Sorrells, 2006). Then, markers were clustered by LD blocks. Clustering was realized by average distance using a cutoff of 1−*R*²c, with *R*²c defined as the 99th percentile of the distribution of unlinked *R*² computed between pairs of markers randomly sampled from different chromosomes. This threshold accounts for a risk of 1% to be in LD by chance. Boundaries of QTL were defined as the minimum and maximum map positions of significant markers belonging to the same LD block.

Finally, we analyzed gene functional annotation in the genomic regions where we found QTLs. Functional annotation was performed by using the genome‐centric portal Ensembl plants (https://plants.ensembl.org). We used the durum wheat cultivar “Svevo” and the wild emmer accession “Zavitan” as reference genomes, as our set of genotypes derived from crosses between durum wheat genotypes and wild emmer accessions. Genes having a known effect on SAS located within the detected QTLs were considered as candidate genes.

## 3 RESULTS

### 3.1 Light effect at the genotype level

**FIGURE 1:**
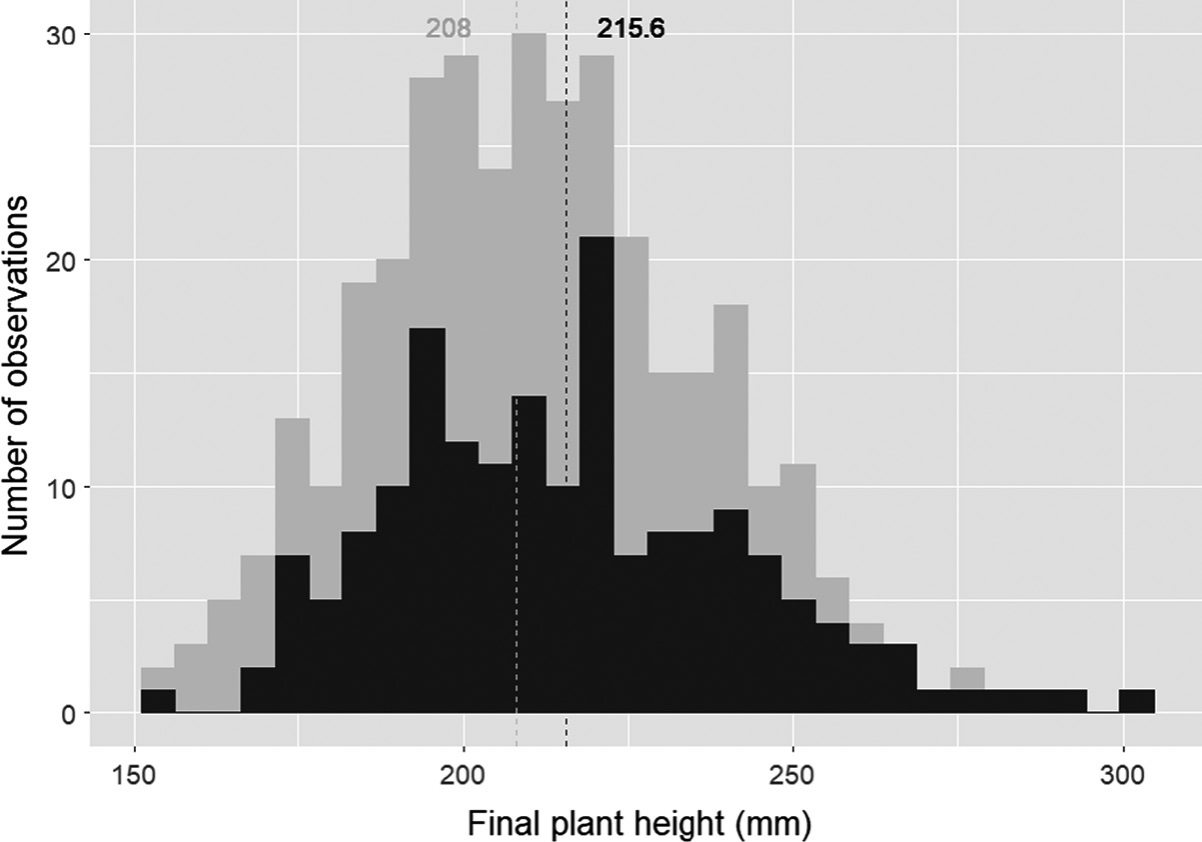
Histograms of plant height BLUE. Dotted line represents means of plant height BLUE in unshaded conditions (low R:FR ratio, light gray) and shaded conditions (high R:FR ratio, dark gray), respectively, from left to right

Plants grew significantly taller in the shaded conditions (+3.4%) than in the unshaded conditions (mean of 215 and 208 mm, and standard error of 27 and 24 mm, respectively) (Figure 1). Broad sense heritabilities for final plant height were moderate in both light quality conditions (36% in the unshaded treatment *versus* 49% in the shaded treatment). Coefficient of genetic variation was about 12% of the means in the unshaded treatment and 13% in the shaded treatment. No significant interaction between light quality and genotype was detected (Table 1). The model included four significant main effects: initial plant height (**η**² = 12%), batch (**η**² = 6%), genotype (**η**² = 26%), and light quality (**η**² = 0.7%).

**TABLE 1:** Variation of plant height in response to light quality

Source of variation	*df*	*F*	*p*‐value	η² (in %)
Initial height	1	236	<0.001	12
Batch	7	15.6	<0.001	6
Genotype	177	2.86	<0.001	26
Light quality	1	12.9	<0.001	0.7
Light quality x Genotype	177	1.00	0.46 (NS)	9
Residuals	1094			56

Note

ANOVA analysis of final plant height. Only the fixed part of the model is reported. η² represents the proportion of variance explained by each effect.

Abbreviations: *df*, degree of freedom; *F*, *F* statistics; NS, nonsignificant.

### 3.2 QTL detection

**FIGURE 2:**
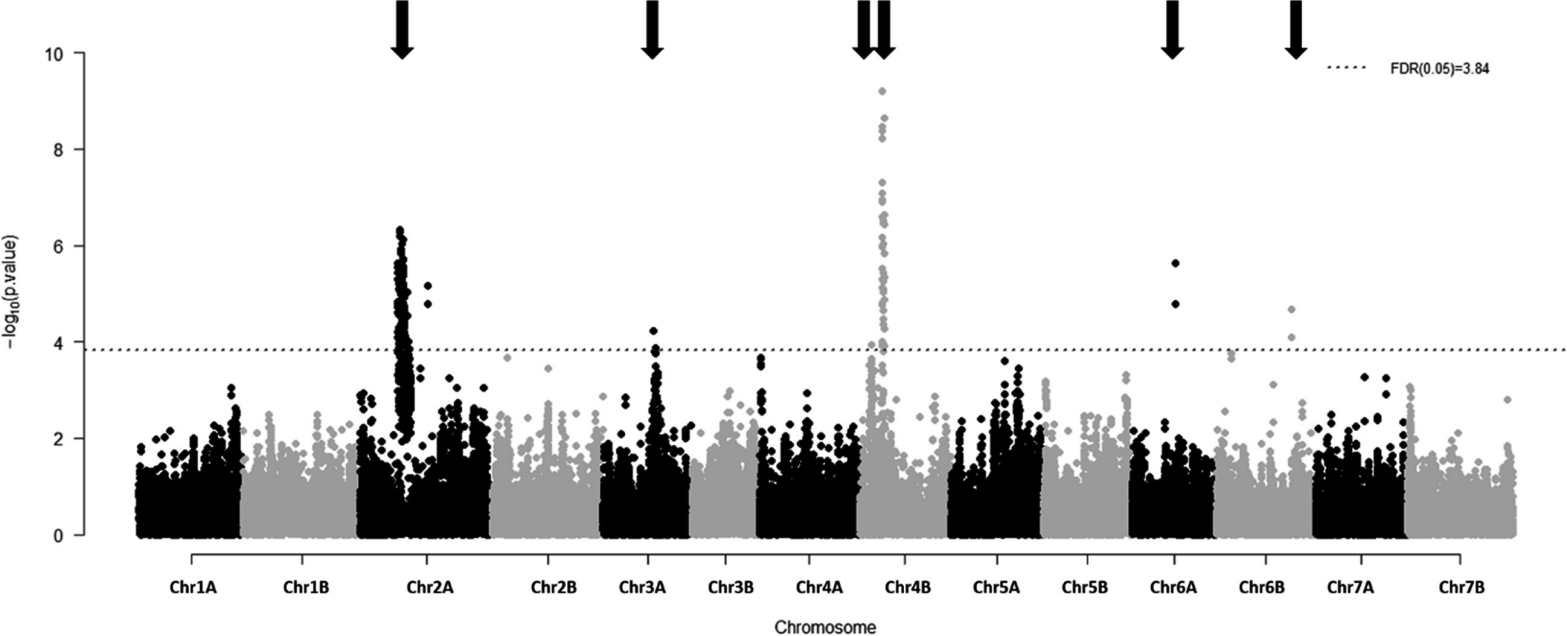
Manhattan plot for plant height. QTLs are indicated with an arrow. QTLs with a significant interactive effect when testing with a sequential type 1 test are represented with a darker arrow. *p*‐value represented in *y*‐axis is *p*‐value resulting from the test of the genetic effect

**FIGURE 3:**
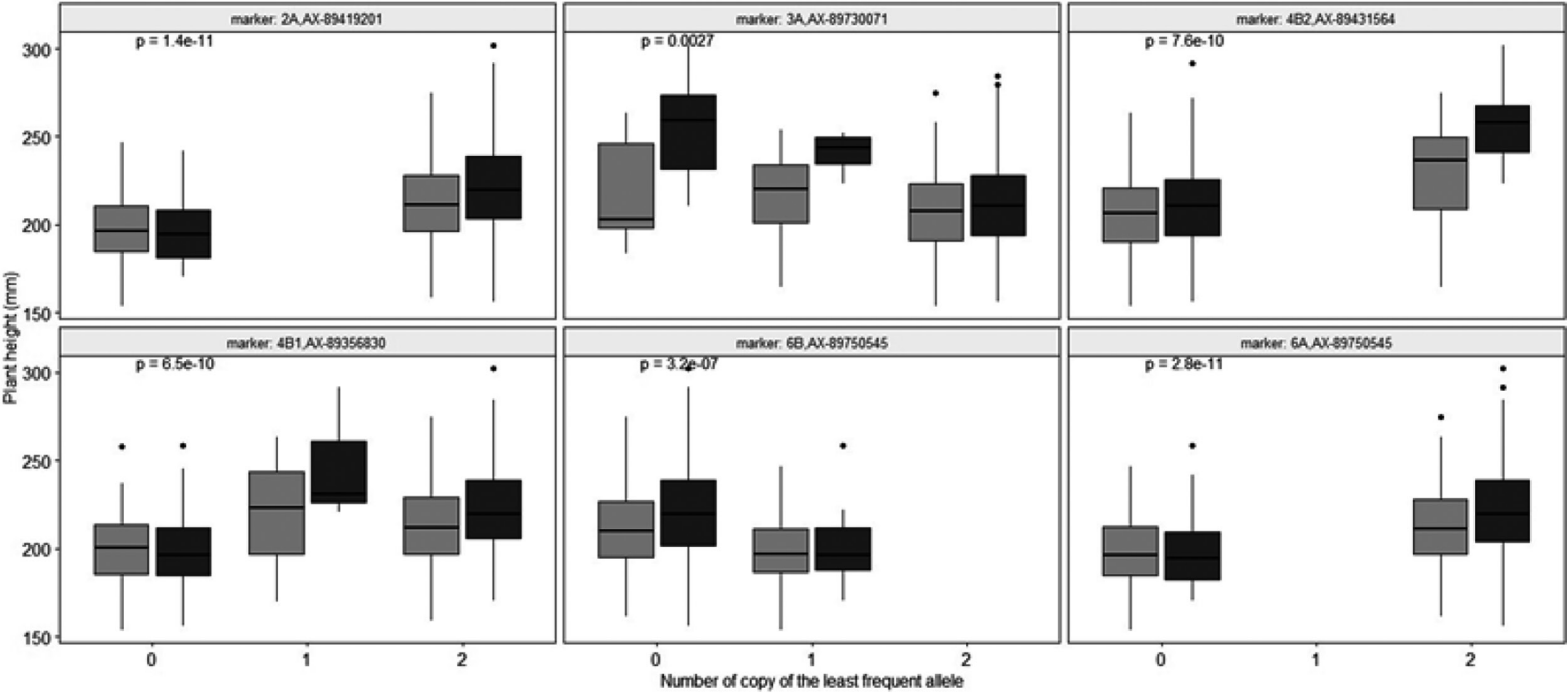
Boxplot of the size effects for each detected QTL. 0, 1, and 2 represent the number of copy of the least frequent allele (note that for QTLs 3A and 6A, few heterozygous genotypes were detected in our panel). *p*‐value indicates the significance of the test from the GWAS accounting for the genetic effect (including marker and marker x light quality effects). Light gray refers to unshaded conditions, whereas dark gray refers to shaded conditions

*p*‐values of the genetic effect of the GWAS study are plotted in a Manhattan plot (Figure 2). Six significant QTLs (2A, 3A, 4B1, 4B2, 6A, 6B) were detected for final plant height (Figure 2; Table 2). Results of the tests for assessing the significance of the fixed part of the mixed model are reported in Table S1. QTLs with the strongest genetic effect were QTL 2A, QTL 3A, and QTL 4B2 (β = 25.4 and β = 16.1 and β = 38.2, respectively; Table 2). Those were also the QTLs with the strongest interactive part (β_inter_ = 5.2, β_inter_ = 11.9 and β_inter_ = 8.5, respectively; Table 2). QTL 3A was characterized by a large part of its genetic effect due to the interaction between the marker and light quality (β_marker_ = 7.4, β_inter_ = 11.9). Because the zero reference taken by the statistical model to compute size effects corresponds to the unshaded treatment and a SNP with two copies of the reference allele, this means that a plant carrying one copy of the least frequent allele at QTL 3A was 7.4 mm taller than a plant carrying two copies of the reference allele when grown in unshaded conditions. Similarly, a plant carrying one copy of the least frequent allele was 19.3 taller (= (β_marker_ + β_inter_)*1 copy = 19.3) than a plant carrying two copies of the reference allele when grown in shaded conditions. Size effects of the interaction term were small for QTL 2A and QTL 4B2 compared with their main size effects (Table 2). Candidate genes with known effect on the SAS were detected in each genomic region harboring QTL 2A, QTL 3A, and QTL 4B2 (Table 2, Table S1). QTLs 4B1, 6A, and 6B showed no significant interaction with light quality with the sequential type I test (LOD_interaction effect_ < 1.3, Table 2). For each of the 6 QTLs, the allele responsible for reduced height also reduced the magnitude of the plant height response to light treatment (Figure 3).

**TABLE 2:** LOD scores and size effects of the six detected QTLs

QTL	Genetic effect		Main effect		Interaction effect	
	LOD score	Size effect	LOD score	Size effect	LOD score	Size effect
2A	6.3*	25.4	5.2*	20.2	1.6*	5.2
3A	4.2*	16.1	2.2	7.4	3.2*	11.9
4B1	3.9*	14.8	3	11.1	1.3	3.7
4B2	9.2*	38.2	7.3*	29.8	2.5*	8.5
6A	4.7*	16.2	4*	15.5	1	2.7
6B	5.6*	22.3	4.8*	18.6	1.3	3.8

Note

The genetic effect representing the joint effect of marker and marker x light quality was estimated from the GWAS, and tested using a false discovery rate (FDR) of 5% (see main text). For each marker, main effect and interaction effect were assessed with a sequential type I test using a threshold of 5%. LOD scores represent log transformed *p*‐values. *Represents significant *p*‐values.

## 4 DISCUSSION

Study of genetic variability is of high importance to understand evolution of a trait within a species. The genetic variability of SAS has received little attention in crop species. Here, we found that plant height response to shade was similar among genotypes. Yet, at the allelic level, we detected three main QTLs associated with SAS, highlighting that genetic variability does exist in our panel.

Plants grew significantly taller in the shaded treatment than in the unshaded one. This result is consistent with the many studies reporting morphological responses of SAS in plants (Franklin & Whitelam, 2005). In our experiment, light quality explained 1% of the total phenotypic variance observed for plant height, meaning that our genotypes did respond only slightly to shade. This could be explained by the fact that measurements were carried out at a very early stage. In agronomic conditions, drop in R:FR ratio at an early stage might not be a good predictor of the strength of future competition as canopy is far from being closed and neighbor plants might only affect slightly the R:FR ratio. This might result in a limited response from the plant early in its life cycle. Other environmental cues that are more dependent on the presence/absence of neighbor plants at early stage might produce stronger plant plasticity at early stage, whereas drop in R:FR ratio might be more informative of stronger competition at later stages and thus result in stronger adaptive plasticity at later stages (Pierik & de Wit, 2014). In addition, the shaded treatment we simulated in our experiment mimicked a change in light mediated by the presence of a neighbor by only modifying the ratio of R:FR light and thus only altering light quality. Therefore, we left aside the molecular mechanisms triggered by a change in light intensity and the amount of blue radiations (Carriedo et al., 2016; Fiorucci & Fankhauser, 2017; Ma & Li, 2019).

At the allelic level, statistical power was higher than at the genotype level, as each allele was observed in several genotypes. We detected six QTLs whose allelic variation was associated with plant height variation. Three of them, QTLs 2A, 3A, and 4B2, showed contrasting effects depending on light quality. Polymorphism and QTLs for plant's height has been identified for most of these six genomic regions (and in particular for QTL 3A and QTL 4B2) in other modern durum wheat populations (Canè et al., 2014) showing that our QTLs are not specific to our population. Interestingly, for all three QTLs showing a significant interactive effect, the allele associated with reduced height was also the one associated with the lowest phenotypic plasticity in response to light quality.

QTL 4B2 is located in a region where there is a major reduced height (Rht) gene, a well‐known dwarfing gene in wheat that has already been shown to have an impact on SAS (Djakovic‐Petrovic et al., 2007). The Rht1 allele in wheat is a mutation in the coding sequence of a DELLA protein involved in the gibberellins (GA) signaling pathways (Peng et al., 1997). Rht1 orthologous genes have been shown to have similar dwarfing properties in barley and rice. In all these species, mutations in Rht genes are associated with modified DELLA proteins that cannot be degraded by GA (Thomas & Sun, 2004), which in turn causes reduced plant growth. Part of the molecular pathway of SAS triggered by variations in the R:FR ratio is GA dependent and involves DELLA proteins. In *Arabidopsis thaliana*, phytochrome B changes conformation under shaded conditions. This stimulates the synthesis of GA that degrades DELLA proteins. This stimulation avoids the inhibition of phytochrome‐interacting factor (PIF) proteins resulting in increased stem elongation. Genotypes bearing Rht1 alleles in wheat have DELLA proteins insensitive to GA‐induced degradation, making them insensitive to variations in light conditions (see Figure 2 of Huber et al., 2021 for detailed mechanisms). Therefore, our results for QTL 4B2 showing that short genotypes are less responsive to light quality are consistent with previous results suggesting that the molecular pathways involved in plant growth and plant growth plasticity might overlap.

QTL 3A was the most sensitive to variation in light quality in our study. Functional annotations identified 202 genes, out of which the DET2 gene was already shown to respond to light conditions (Vandenbussche et al., 2005). This gene, involved in the second step of the biosynthesis of brassinosteroids, is necessary for the production of BZR1, a transcriptional factor integrated in the light signaling pathway (Oh et al., 2012). This transcriptional factor is repressed under unshaded conditions, thus limiting plant growth. Nonfunctional DET2 gene reduces the production of BZR1 leading to dwarf genotypes insensitive to light variation (see Fridman & Savaldi‐Goldstein, 2013 for a review). Interestingly, the locus BZR1 was located in the QTL 2A. Such genetic correlation between the trait and its plasticity in response to light conditions has also been documented for the Tb1 gene involved in the tillering ability of maize (Lukens & Doebley, 1999). Transcription of this gene is induced by shaded conditions and increases apical dominance, thus reducing tillering ability. In modern germplasm, mutation in the regulatory sequence of this gene induces constant overexpression of the gene, leading to strong apical dominance and thus a single culm phenotype in both shaded and unshaded conditions (Lukens & Doebley, 1999). Similarly to light‐mediated plant height plasticity induced by DELLA proteins, single culm phenotypes in maize are insensitive to light (Lukens & Doebley, 1999).

In our study, the QTL analysis of SAS highlights a positive genetic correlation between plant height value and plant height plasticity. Such genetic correlation can arise either from a pleiotropic effect where the same gene(s) affect both the trait and its plasticity, or from genetic linkage between trait‐associated gene(s) and plasticity‐associated genes. Positive genetic correlation on trait values and trait plasticity have already been documented in the literature (Lafuente & Beldade, 2019; Pfennig, 2021). For instance, most loci affecting flowering time across environments also affect flowering time environmental plasticity in *Arabidopsis thaliana* (reviewed in Pfennig, 2021). Such a positive genetic correlation between trait value and trait plasticity implies that any selective pressure on a given trait might affect the magnitude of its plasticity. This suggests that plasticity might be a by‐product of selection on the trait itself (Lafuente & Beldade, 2019). Current view on the genetic mechanisms underlying plasticity can explain these patterns without excluding the existence of an independent selection on plasticity. Indeed, many studies support the view that genetic variants that regulate key master regulators interact with each other and with the environment (reviewed in Pfennig, 2021). The transcriptional hub of the DELLA protein complex is a good example of such phenomenon in plants (Blanco‐Touriñán et al., 2020). Thus, any nonsynonymous mutation in sequences coding for proteins of these complexes will affect both trait value across environments and trait plasticity. In contrast, other studies have found independent genetic determinisms between trait value and trait plasticity (Lafuente & Beldade, 2019; Pfennig, 2021). In these studies, authors might have identified the genetic determinism of environmental sensors affecting traits only in specific environments. This would suggest that trait plasticity could evolve independently from the trait itself (Sommer, 2020). In most studies as in ours, distinguishing between pleiotropic effects and linkage disequilibrium effects is not possible. To go a step further, it would be interesting to test the QTLs in populations with different linkage disequilibrium structure. Additionally, provided the development of new plant material, we could perform a functional analysis of these QTLs see for instance (Lafuente et al., 2018) to strengthen the previous discussion.

Positive genetic correlation between height and height plasticity in response to light conditions has been documented as an adaptive response in many ecosystems (Henry & Aarssen, 2001b; Schmitt et al., 2003). For instance, the herbaceous species *Impatiens capensis* does express SAS in herbaceous communities where it is among the tallest species that might benefit from SAS to outcompete its neighbors. In contrast, this species does not show any SAS when growing in woodlands, where it is among the smallest species in the community (Schmitt et al., 2003). Interestingly SAS has also been documented in trees that represents the upper layer of forest ecosystems (Henry & Aarssen, 2001a). This suggests that SAS‐mediated height plasticity only occurs in the tallest species of the community in natural ecosystems. In agrosystems, before the Green Revolution in France, many wheat farmers were still growing heterogeneous landrace populations harboring large genetic variability (Bonnin et al., 2014). In such fields, we thus expect light intensity to decrease exponentially within the canopy (Moreau et al., 2012). As described in natural ecosystems, alleles inducing high plasticity in height in response to light conditions might be associated with tall alleles as plastic alleles might increase fitness and competitive ability of tall genotypes only.

Since the Green Revolution, numerous dwarfing genes have been introgressed in the modern germplasm (Hedden, 2003). Initially introduced to reduce lodging in high nitrogen conditions, dwarfing alleles induce both higher harvest index and lower plant competition between plants (Donald, 1968). These alleles have become more frequent in the modern germplasm. For instance, the vast majority of durum wheat elite varieties produce the mutated DELLA protein at the Rht locus located on the 4B chromosome (Clarke et al., 2012). Directional selection for shorter plants might have thus reduced the SAS mediated by plant height plasticity in modern germplasm, making modern cultivars selected for growing at high density less sensitive to neighbors. Interestingly, nonfunctional DELLA proteins as those coded by the Rht semi‐dwarf allele have been documented to reduce phenotypic plasticity on many other traits in response to other environmental signals such as low temperatures, salty conditions, and drought (Van De Velde et al., 2017).

Faced with the need to develop more sustainable agriculture with reduced chemical inputs, we might expect plant breeding to consider SAS as a trait to integrate in breeding schemes. During the Green Revolution, drawbacks of short genotypes (reduced competitive ability toward weeds, inability to escape from soil‐borne diseases, etc.) were compensated by the use of chemicals and the generalization of inbred lines. This has cleared the way for the massive introgression of the Rht1 dwarf allele. In a context of reduced pesticide use, weed control by other means than herbicides will become important to implement. Genotypes growing taller in the early stage of the cycle might thus be of interest to limit weed development (Weinig, 2000). However, at later stages, shade avoidance is also known for preventing tiller formation, changing leaf position to more vertical orientation, and reducing leaf to stem biomass ratio (Kebrom & Brutnell, 2007a). This promotes a more open canopy allowing higher light penetration inside the canopy (Pantazopoulou et al., 2021 for *Arabidopsis thaliana*) and thus greater weed development in agro‐systems. SAS might thus become a breeding target especially at early stage, where crops’ main competitors are weeds. In this context, it could be relevant to decouple SAS and plant height to breed for dwarf plants (with high harvest index) with strong SAS at early stages. Our results show that genetic polymorphism does exist at QTLs that mainly affect SAS at early stages rather than plant height. This is the case of QTL 3A. More generally, with increasing regulation of pesticide use and increasing environmental variability arising from global change, our results highlight the need to re‐assess the relevance of some Green Revolution genes to tackle agricultural challenges.

## CONFLICT OF INTEREST

The authors declare no conflict of interest.

## Supporting information

Table S1

## Data Availability

The data are available on request at michel.colombo@inrae.fr [Correction added on 2nd March 2022, after first Online publication: Data Availability Statement has been updated in this version]
