# Supplementary material for: A genome‐wide analysis suggests pleiotropic effects of Green Revolution genes on shade avoidance in wheat
Source: Evol Appl. 2022 Feb 19;15(10):1594–604. doi: 10.1111/eva.13349 (PMC9624089; doi:10.1111/eva.13349)
Supplement: Supplementary file 1 — Table S1 [file EVA-15-1594-s001.docx]

Supplementary materials

**Table S1.** **Functional annotation of the 3 detected QTLs having a significant marker X light quality effect**

| **Marker** | **QTL** | **Position** | **Number of annotated genes in NCBI** | **Candidate genes** |
| --- | --- | --- | --- | --- |
| AX-89419201 | 2A | 138010706 | 1268 | CKII  BES1/BZR1 |
| AX-89730071 | 3A | 603980840 | 202 | DET2 |
| AX-89431564 | 4B2 | 29280812 | 68 | Rht1 / DELLA protein |

Note: position along the chromosome is expressed in number of base pairs. Candidate genes correspond to genes located within the detected QTL and whose impact on the SAS have been reported in the literature.
